# Inhibitory effects of *Jasminum grandiflorum* L. essential oil on lipopolysaccharide-induced microglia activation-integrated characteristic analysis of volatile compounds, network pharmacology, and BV-2 cell

**DOI:** 10.3389/fphar.2023.1180618

**Published:** 2023-08-04

**Authors:** Jingya Lu, Xiaoyan Zeng, Yanping Feng, Siyi Li, Yun Wang, Youlin Liu, Feilong Chen, Zhenfeng Guan, Tiantian Chen, Fenghuan Wei

**Affiliations:** ^1^ School of Traditional Chinese Medicine, Southern Medical University, Guangzhou, China; ^2^ NMPA Key Laboratory of Rapid Drug Detection Technology, Guangdong Institute for Drug Control, Guangzhou, China; ^3^ Guangdong Provincial Key Laboratory of Chinese Medicine Pharmaceutics, Guangzhou, China

**Keywords:** *Jasminum grandiflorum* L. flowers, HS-SPME-GC–MS/MS, essential oils, network pharmacology, microglia activation

## Abstract

Neuroinflammation is considered to have a prominent role in the pathogenesis of Alzheimer’s disease (AD). Microglia are the resident macrophages of the central nervous system, and modulating microglia activation is a promising strategy to prevent AD. Essential oil of *Jasminum grandiflorum* L. flowers is commonly used in folk medicine for the relief of mental pressure and disorders, and analyzing the volatile compound profiles and evaluating the inhibitory effects of *J. grandiflorum* L. essential oil (JGEO) on the excessive activation of microglia are valuable for its application. This study aims to explore the potential active compounds in JGEO for treating AD by inhibiting microglia activation-integrated network pharmacology, molecular docking, and the microglia model. A headspace solid-phase microextraction combined with the gas chromatography–mass spectrometry procedure was used to analyze the volatile characteristics of the compounds in *J. grandiflorum* L. flowers at 50°C, 70°C, 90°C, and 100°C for 50 min, respectively. A network pharmacological analysis and molecular docking were used to predict the key compounds, key targets, and binding energies based on the detected compounds in JGEO. In the lipopolysaccharide (LPS)-induced BV-2 cell model, the cells were treated with 100 ng/mL of LPS and JGEO at 7.5, 15.0, and 30 μg/mL, and then, the morphological changes, the production of nitric oxide (NO) and reactive oxygen species, and the expressions of tumor necrosis factor-α, interleukin-1β, and ionized calcium-binding adapter molecule 1 of BV-2 cells were analyzed. A total of 34 compounds with significantly different volatilities were identified. α-Hexylcinnamaldehyde, nerolidol, hexahydrofarnesyl acetone, dodecanal, and decanal were predicted as the top five key compounds, and SRC, EGFR, VEGFA, HSP90AA1, and ESR1 were the top five key targets. In addition, the binding energies between them were less than −3.9 kcal/mol. BV-2 cells were activated by LPS with morphological changes, and JGEO not only could clearly reverse the changes but also significantly inhibited the production of NO and reactive oxygen species and suppressed the expressions of tumor necrosis factor-α, interleukin-1β, and ionized calcium-binding adapter molecule 1. The findings indicate that JGEO could inhibit the overactivation of microglia characterized by decreasing the neuroinflammatory and oxidative stress responses through the multi-compound and multi-target action modes, which support the traditional use of JGEO in treating neuroinflammation-related disorders.

## 1 Introduction

Alzheimer’s disease (AD) is the most common form of neurodegenerative disease, estimated to contribute 60%–70% of all cases of dementia worldwide, which affects more than 50 million people globally, and its incidence is increasing as the elderly population increases. Among the numerous pathogeneses of AD, neuroinflammation and oxidative stress are considered to have prominent roles in the pathogenesis of AD (Chuang et al., 2015; Leng and Edison., 2021). As the primary players in neuroinflammation and the innate immune cells in the central nervous system (CNS), microglia account for 5%–20% of all glial cells in the mammalian adult brain and are marked by the excessive production of proinflammatory cytokines, including interleukin-1β (IL-1β), tumor necrosis factor-α (TNF-α), and interleukin-6 (IL-6), and small-molecule messengers, including nitric oxide (NO) and reactive oxygen species (ROS) (DiSabato et al., 2016; Ndoja et al., 2020). Upon overexpression of these inflammatory factors, the role of neuroinflammation will change from an immune defense protection function on the CNS to attacking healthy neurons, thereby resulting in neuronal death and, eventually, the induction of degenerative diseases. In the context of neurodegenerative diseases, neuroinflammation tends to be a chronic process that fails to resolve by itself and is considered a vital driver of neurodegenerative diseases (Leng and Edison., 2021). Therefore, suppressing microglial activation can have beneficial effects in preventing neuroinflammation, thus preventing AD at an earlier stage and delaying the progression of AD.

Due to the complexity of AD pathogenies, available drug treatments only target the symptoms and do not halt the progression of AD. The use of multi-compound herbal medicine and natural compounds as alternative intervention strategies is rapidly rising, which also presents promising prospects (Angeloni et al., 2019; Liang et al., 2022). As of now, encouraging results by using essential oils (EOs) for treating AD have been published. As shown in Table 1, EOs from different sources significantly inhibited the excessive production of proinflammatory cytokines, oxidative stress, and AD-related enzyme activities and even improved the cognitive abilities of AD animal models. Taken together, exploring valuable EOs from herbs based on folk application and experimental studies for counteracting pathophysiological processes of AD would be valuable and promising.

**TABLE 1:** Effects of essential oils recently reported on preventing AD-related symptoms.

Materials	Models	Pharmacological actions	References
*Monarda didyma* L.	D-galactose-induced mice	Improved learning and memory impairment of aging mice via Nrf2 and MAPK pathways	Guo et al. (2022)
Lemon	APP/PS1 and wild-type mice	Improved memory of AD animals by reducing AChE levels and elevating BDNF, PSD95, and synaptophysin	Liu et al. (2020)
*Pinus halepensis*	Aβ_42_-induced rats	Improved learning and memory impairment of rats by attenuating Aβ toxicity and neuronal dysfunction	Postu et al. (2019)
*Tetraclinis articulata*	Aβ_42_-induced rats	Improved learning and memory impairment of rats by retrieving AChE activity and oxidant status	Sadiki et al. (2019)
Garlic	Healthy mice	Inhibited the activities of BACE, AChE, and BChE	Yoshioka et al. (2021)
Lavender	PC12 cells	Inhibited neurotoxicity by improving cell viability, reducing ROS production, and activating caspase-3	Caputo et al. (2021)
*Polygonum hydropiper*	APP transgene animals	Augmented motor and coordination abilities of animals by declining AChE and BChE activities	Tong et al. (2020)
*Schisandra chinensis*	Aβ_42_- and LPS-induced mice	Improved cognitive ability of mice by suppressing the production of TNF-a, IL-6, and IL-1β and p38 activation in the hippocampus	Xu et al. (2019)

The flowers and flower buds of *Jasminum grandiflorum* L. (family Oleaceae) (wfo-0000813821, the plant name has been checked with http://www.worldfloraonline.org on 20 May 2023) are commonly used for treating hepatitis, stomatitis, psychiatric disorders, and other disorders in Southeast Asia (Ferreres et al., 2014; Zhang et al., 2021). So far, studies on extracts from different parts of *J. grandiflorum* L. plants reported (showed in Table 2) results that expound some potential active compounds, pharmacological effects, and action mechanisms of *J. grandiflorum* L. plants in treating various disorders; however, most of the publications are on flavonoids and organic acids. Considering that *J. grandiflorum* L. essential oil with a strong sweet-smelling fragrance is widely used in aromatherapy for relieving mental stress, we preliminarily identified 30 components in essential oils extracted from *J. grandiflorum* L. by the steam distillation method and identified by gas chromatography–mass spectrometry (GC–MS) (Wei et al., 2015). Among them, the major components were phytol amounting to 25.77%, nerolidol amounting to 12.54%, and isophytol amounting to 12.42%; these three main compounds not only possess anti-inflammatory and antioxidant activities but also have been approved as food flavors by the U.S. Food and Drug Administration or European Union (Hultqvist et al., 2006; Hsu et al., 2012; De Carvalho et al., 2018; Albinsaad et al., 2021; Arunachalam et al., 2021; Vahdati et al., 2022). Thus, exploring the anti-neuroinflammatory effects of JGEO would be valuable. Headspace solid-phase microextraction (HS-SPME) combines sampling, analyte isolation, and enrichment in one step, and the true component profiles are analyzed without the interference of extraction temperature; thus, HS-SPME-GC–MS/MS is widely used to analyze the compound profiles in EOs (Mascrez et al., 2020; Song et al., 2021; Baky et al., 2022; Su et al., 2022). Though fresh flowers of *J. grandiflorum* L. were analyzed by SPME/GC–MS (Issa et al., 2020), considering the more convenient method for preservation and more often for usage of dried flowers in folk medicine and the inevitable impact of the drying process on compounds in EOs (Qiu et al., 2020), expounding the compound profiles of dried flowers of *J. grandiflorum* L. by HP-SPME/GC–MS is helpful to clarify its key compounds in treating mental disorders.

**TABLE 2:** Pharmacological actions and active compounds of *Jasminum grandiflorum* L. plants.

Parts	Extract solvent	Pharmacological actions	References
Flowers	Methanol	Showed significant nephroprotective effects on cisplatin-induced rats	Alqahtani et al. (2022)
Flowers	Essential oil	Inhibited viral-infected and cancer cells	Mansour et al. (2022)
Flowers	Petroleum ether, ethyl acetate, and n-butanol	Alleviated hepatic toxicity induced by CCl_4_	Sun et al. (2022)
Flowers	Petroleum ether, ethyl acetate, and n-butanol	Alleviated gastric mucosal ulceration	Zhang et al. (2021)
Flowers	Petroleum ether, ethyl acetate, and n-butanol	Alleviated TPA-induced mouse skin inflammation	Li et al. (2020)
Flowers	Water and methanol	Exhibited antioxidant activities and inhibited CNS-related enzymes	Ferreres et al. (2014)
Flowers	Aqueous extract	Decreased NO levels in RAW 264.7 macrophages	Oliveira et al. (2017)
Flowers	95% ethanolic	Improved the antioxidant defense system in DMBA-treated rats	Kolanjiappan and Manoharan, (2005)
Leaves	Leaf powder	Accelerated wound healing	Mortazavi et al. (2020)
Aerial parts	Methanolic extract	Inhibited the inflammation of rheumatoid arthritis in mice	El-Shiekh et al. (2021)
Aerial parts	Dichloromethane and n-butanol	Displayed anthelmintic activity	Hussein et al. (2021)
Aerial parts	Methanolic extract	Showed ACE inhibition and antioxidant activity *in vitro*	El-Shiekh et al. (2020)

Therefore, in this study, JGEO treatment of mental disorders by suppressing neuroinflammation and oxidative stress via inhibiting the overactivation of microglia was hypothesized. To test this hypothesis, analyzing volatile compound profiles of *Jasminum grandiflorum* L. by HS-SPME/GC–MS, predicting targets and target pathways of anti-inflammation using network pharmacology and molecular docking, and validating the effects using the LPS-induced BV-2 cell model were carried out, respectively. This study will provide novel ideas for using *Jasminum grandiflorum* L. to prevent neurodegenerative diseases associated with neuroinflammation.

## 2 Materials and methods

### 2.1 Chemicals and reagents

Iba1 rabbit mAb (ab178847, GR3229566-23), TNF-α rabbit mAb (ab234437, GR284782-13), and IL-1β rabbit mAb (ab234437, GR3354222-10) were bought from Abcam (Cambridge, United Kingdom). Dulbecco’s modified Eagle’s medium (DMEM, 8122043) and penicillin–streptomycin (P.S, 15140-122) were bought from Gibco (Thermo Fisher Scientific, United States). Lysis buffer for Western blotting and IP, phenylmethanesulfonyl fluoride (PMSF, 020421210524), DAPI staining solution (030521210706), a nitric oxide assay kit (022421210729), a BCA kit (091922230203), and the Immunol Fluorescence Staining Kit with FITC-labeled goat anti-rabbit IgG (070121210917) were bought from Beyotime Biotechnology (Shanghai, China). The ROS Fluorometric Assay Kit (21091440) was bought from Elabscience Biotechnology Co., Ltd. (Wuhan, China). Fetal bovine serum (FBS, 12A125) was bought from ExCell Bio (Shanghai, China). Lipopolysaccharide (LPS, *Escherichia coli* 055:B5,21145395) was bought from Biosharp (Guangzhou, China). β-Actin rabbit mAb (4970T, 15) and anti-rabbit IgG and HRP-linked antibody (7074P2, 28) were bought from Cell Signaling Technology (Danvers, MA, United States). The CCK8 assay kit (Z2040220204C) was bought from Zeta Life (United States), and BV-2 cells were obtained from BeNa Culture Collection, China.

### 2.2 *Jasminum grandiflorum* L. Materials

*Jasminum grandiflorum* L. flowers were purchased from Zhixin Chinese Pharmaceutical Co., Ltd., Guangdong Province, China. Samples were identified by Professor Hongwei Zhang (Department of Medicinal Plants and Pharmacognosy, Southern Medical University, Guangzhou, China) and were kept in a desiccator.

### 2.3 HS-SPME procedure

For this procedure, 0.5 g of *Jasminum grandiflorum* L. was sealed in a 20 mL headspace screw-top vial with a magnetic screw cap and PTFE seal (Thermo Scientific, Germany), respectively. The extraction was successively performed using a 75 µm carboxen/polydimethylsiloxane (CAR/PDMS) fiber (Supelco, United States) at 50°C, 70°C, 90°C, and 100°C for 50 min, respectively. After each extraction, the fiber was manually inserted into a Trace GC–MS system equipped with an Ultra GC and a quadrupole MS for 5 min to desorb analytes. The fiber was preconditioned before use according to the supplier’s instruction and cleaned for 5 min between injections to prevent cross-contamination.

### 2.4 GC–MS analysis

The GC–MS analysis was performed on a gas chromatograph (Trace 1310, Thermo Scientific, United States) equipped with a single quadrupole mass spectrometer (ISQ LT, Thermo Scientific, United States). The separation was conducted using a DB-5 capillary column (30 m × 0.25 mm i. d., film thickness 0.25 μm, Agilent). The oven temperature program was initially set at 65°C for 2 min, warmed to 140°C at 5°C/min for 10 min, then heated to 180°C at 3°C/min for 10 min, and finally, increased to 250°C at 20°C/min for 5 min. The electron impact (EI)-MS was operated at 70 eV, and the scan range (rate) was set as 45–500 amu (0.20 s per scan). The injector, MS transfer line, and ion source were kept at 220, 280, and 230°C, respectively. The splitless injection mode was used for analysis. Identification of volatiles was achieved by comparing the mass spectra and RI values with those of the known compounds in the standard NIST 11 library and WILEY275 library.

### 2.5 Extraction of *Jasminum grandiflorum* L. flower essential oil

JGEO was extracted from 200 g of *Jasminum grandiflorum* L*.* by the hydro-distillation method: flowers were immersed in six times water and were extracted for 3 h in a volatile oil extractor. Then, the JGEO was restored at 4°C in a refrigerator for further use.

### 2.6 Network pharmacology

#### 2.6.1 Potential targets of compounds in JGEO and inflammation

The potential target proteins of the compounds detected in *Jasminum grandiflorum* L. to *Homo sapiens* were searched from the Swiss TargetPrediction database (http://www.swisstargetprediction.ch/). The disease targets related to neuroinflammation and inflammation were searched in GeneCards (https://www.genecards.org/). The naming normalization of the targets was carried out using the UniProt database (https://www.uniprot.org/). The potential targets of compounds predicted by the Swiss TargetPrediction database were mapped to inflammation-related targets using Venny 2.1.0 (https://bioinfogp.cnb.csic.es/tools/venny/), and then, their intersection genes were considered to be the effective targets of JGEO relating to inflammation and neuroinflammation.

#### 2.6.2 Protein–protein interaction network

The STRING database (https://string-db.org/) was used to conduct protein–protein interaction (PPI), and the interaction networks of PPIs were constructed by Cytoscape 3.6.0 software.

#### 2.6.3 GO and KEGG pathway enrichment analyses

The Metascape database (https://metascape.org/) was used to carry out the enrichment analysis of KEGG pathways and GO biological processes. The KEGG biological pathway enrichment analysis selected the pathways with a *p*-value ≤0.01, whereas the top 10 pathways with count values were selected. A GO enrichment analysis was carried out, including cellular components, molecular functions, and biological processes. Using the Bioinformatics Platform (http://www.bioinformatics.com.cn/), the visualization analysis was performed to create the bubble chart and histogram.

#### 2.6.4 Construction of a key compound–target–pathway network

The topological attributes of the network were analyzed using the network analyzer function in Cytoscape 3.6.0 to calculate three important topological parameters: degree, betweenness centrality, and closeness centrality. In the network, nodes represented compounds and proteins related to neuroinflammation, and edges represented compound–target or disease–target interactions.

#### 2.6.5 Molecular docking

The 2D structures of the main active compounds were searched from the PubChem database, and then, Chem 3D was used to minimize the energy of the compounds. All the abovementioned active compounds were imported into AutoDockTools. The ternary crystal structures of the main target proteins were searched from the PDB database (http://www.rcsb.org/), where water molecules were removed and hydrogen atoms were added. The docking activity was evaluated according to the docking calculation results. The protein–ligand docking was achieved by a grid box for covering the binding sites of enzymes. Evaluation of docking results was sorted via predicted binding energy (kcal/mol). The best different poses of ligand and receptor interactions were recorded. Among the results, the highest binding scores of ligand pose and interactions were displayed.

### 2.7 LPS-induced BV-2 cell model

#### 2.7.1 Cell culture and treatment

*In vitro* models have an increasingly important role in dissecting and targeting the causal mechanisms underlying AD (Blanchard et al., 2022). Here, BV-2 cells were chosen as cell models to evaluate the effects of JGEO. The immortalized murine microglia cell line BV-2 was cultured at 37°C in a 5% CO_2_ humidified incubator in Dulbecco’s modified Eagle’s medium supplemented with 10% fetal bovine serum and 100 U/mL of penicillin–streptomycin. The culture medium was changed to a fresh medium every 2 or 3 days, and when the cells reached confluence, they were split into new flasks or used immediately for the experiments. In each experiment, BV-2 cells were cultured with or without 7.5, 15, and 30 μg/mL of JGEO for 4 h and then were treated with 100 ng/mL of lipopolysaccharide (LPS) for 24 h. The cells in the blank control group were neither treated with EOs nor stimulated by LPS.

#### 2.7.2 Cell viability assay and cell morphology observation

BV-2 cells were seeded in a 96-well plate (5×10^3^ cells/mL) and treated by following the procedure mentioned in “Section 2.7.1.” BV-2 cell viability was determined by using a CCK8 assay kit according to the manufacturer’s instructions, and cell viability was calculated from the optical density obtained using an ELISA reader. Cell morphologies were observed under a light microscope.

#### 2.7.3 NO assay

BV-2 cells were seeded in a 96-well plate (8 × 10^3^ cells/mL) and treated by following the procedure mentioned in “Section 2.7.1.” NO levels were assessed indirectly by measuring the accumulated nitrite levels in the supernatant using the nitric oxide assay kit with classical Griess reagents. Then, 50 μL of supernatant from each sample was extracted and processed according to the manufacturer’s protocol. A standard curve was generated in the range between 0 and 20 μM using nitrite as a standard sample. The nitrite concentration per sample was determined using the external calibration curve.

#### 2.7.4 Western blot assay

BV-2 cells were seeded at 5 × 10^5^ cells per well in six-well plates and treated by following the procedure mentioned in “2.7.1 Section.” Whole-cell proteins were homogenized with 150 μL lysis buffer for WB/IP containing 1 mM phenylmethanesulfonyl fluoride for 30 min s under 4°C after washing with PBS solution, and then, cell debris was cleared by centrifugation for 10 min at 10,000 g under 4°C. Protein concentration was determined using a BCA kit. Equal amounts of proteins were separated using 12% SDS-PAGE gels, and the separated proteins were transferred onto 0.45 μm PVDF membranes. After blocking with 5% nonfat dried milk for 2 h, the membranes were incubated with TNF-α rabbit mAb (1:1,000, Abcam), IL-1β rabbit mAb (1:1,000, Abcam), and β-actin rabbit mAb (1:1,000, CST) overnight at 4°C. Next, the membranes were incubated with the secondary antibody (anti-rabbit IgG, HRP-linked antibody, 1:10000, CST) for 1 h. The density of each immunoblot was scanned using the ImageJ software, and the ratios of target protein to β-actin were calculated and used to conduct statistical analysis.

#### 2.7.5 ROS assay

BV-2 cells were seeded at 5 × 10^5^ cells per well in six-well plates and treated by following the procedure mentioned in “Section 2.7.1.”. Then, the cells were washed with detergent once according to the instructions of the ROS Fluorometric Assay Kit. Cells except the negative control group were incubated with the probe of 2, 7-dichlorofuorescin diacetate (DCFH-DA, 10 μM) for 1 h at 37°C in the dark to assess intracellular ROS. Samples were measured at 488 nm excitation wavelength on a fluorescence microscope in the same parameters, and ROS levels were quantitated by the ImageJ software.

#### 2.7.6 Immunofluorescence

BV-2 cells were seeded at 1 × 10^5^ cells per well in a 24-well plate where 14 mm round coverslips had been placed on each well. After treatment, the cells were washed with pre-warmed PBS twice, fixed with methanol for 20 min, and washed with TBSTX containing 0.1% Triton X-100 three times. Then, the cells were blocked with 5% BSA diluted by TBSTx for 1 h at room temperature. Incubations with Iba1 rabbit mAb (1:100) were carried out overnight at 4°C. Finally, cells stained for Iba1 were incubated with FITC-labeled secondary antibody (1:1,000, 1 h, room temperature, at dark). Following washing with TBSTx, microglia nuclei were counterstained with DAPI for 5 min. All coverslips were washed three times with TBSTx and sealed using an anti-fluorescent quenching agent. Fluorescence microscopy imaging was performed, and quantification of fluorescence intensity was performed using ImageJ.

### 2.8 Statistical analysis

Results are expressed as the mean ± standard error of the mean (SEM). Statistical analysis was performed using the IBM SPSS20 software. Statistical significance of differences among multiple groups was determined by one-way ANOVA followed by the LSD test or evaluated by Welch ANOVA followed by Dunnett’s T3, while heterogeneity of variance was measured. A *p* < 0.05 was considered significant.

## 3 Results and discussion

### 3.1 Volatile compound characteristics of *Jasminum grandiflorum* L. by HS-SPME-GC–MS

**FIGURE 1:**
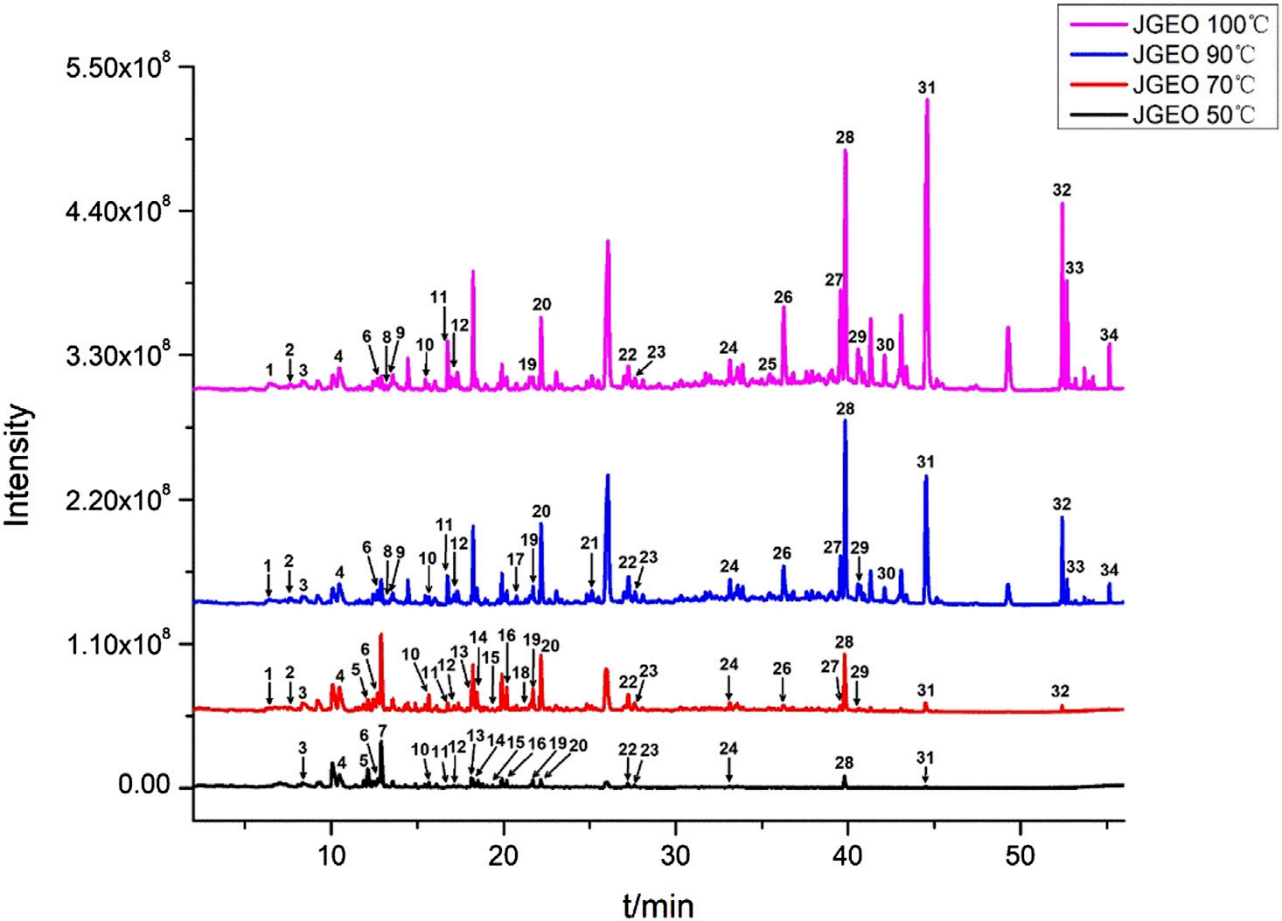
Volatile compounds in *Jasminum grandiflorum* L. flowers detected at different extract temperatures using a gradual warming HS-SPME procedure with GC–MS/MS.

To clarify the volatile compound profiles of *Jasminum grandiflorum* L. without the influence of reflux heating and volatilization loss, a gradual warming HS-SPME procedure was set up at 50°C, 70°C, 90°C, and 100°C for 50 min, respectively. All GC–MS/MS data were integrated manually to avoid the interference of base peaks, and the peaks were matched with the parameters of qual ≥90 from the standard NIST 11 library and qual ≥700 from the WILEY275 library, respectively. As shown in Figure 1 and Table 3, a total of 34 volatile compounds in *J. grandiflorum* L. were identified, which were categorized into 10 different classes, namely, sesquiterpenoids, monoterpenes, diterpenes, aromatic alcohols, phenols, aromatic aldehydes, aromatic esters, aliphatic aldehydes, fatty alcohol, and alkanes. In addition, diterpenes, aliphatic aldehydes, aromatic alcohols, sesquiterpenoids, and phenols are amounted as the major categories in *J. grandiflorum* L.

**TABLE 3:** Volatile compounds of *Jasminum grandiflorum* L. detected by HS-SPME/GC–MS.

No.	R_t_/min		Compounds	Categories	Formula	CAS	Area, %			
							50 °C	70 °C	90 °C	100 °C
1	6.37		Benzaldehyde	Aromatic aldehydes	C_7_H_6_O	100-52–7	-	1.02	0.63	0.94
2	7.59		(E,E)-2,4-heptadienal	Fatty aldehydes	C_7_H_10_O	4313-03–5	-	0.86	0.44	0.33
3	8.34		Benzyl alcohol	Aromatic alcohols	C_7_H_8_O	100-51–6	3.02	1.18	0.98	0.88
4	10.46		Phenylethyl alcohol	Aromatic alcohols	C_8_H_10_O	60-12–8	8.01	4.96	2.35	1.70
5	12.12		Levomenthol	Monoterpenes	C_10_H_20_O	2216-51–5	6.29	1.43	-	-
6	12.71		Methyl salicylate	Phenols	C_8_H_8_O_3_	119-36–8	3.68	2.58	0.93	0.49
7	12.90		Decanal	Fatty aldehydes	C_10_H_20_O	112-31–2	13.74	-	-	-
8	13.17		(E,E)-2,4-nonadienal	Fatty aldehydes	C_9_H_14_O	5910-87–2	-	-	0.15	0.14
9	13.45		2-Phenoxyethanol	Aromatic alcohols	C_8_H_10_O_2_	122-99–6	-	-	0.21	0.20
10	15.66		Undecanal	Fatty aldehydes	C_11_H_22_O	112-44–7	0.99	1.02	0.29	0.16
11	16.76		Methyl anthranilate	Aromatic esters	C_8_H_9_NO_2_	134-20–3	0.43	0.84	1.26	1.35
12	17.12		Eugenol	Phenylpropanoids	C_10_H_12_O_2_	97-53–0	0.52	0.57	0.44	0.35
13	18.12		Tetradecane	Aliphatic alkanes	C_14_H_30_	629-59–4	1.62	1.17	-	-
14	18.43		Dodecanal	Fatty aldehydes	C_12_H_24_O	112-54–9	0.68	1.21	-	-
15	19.36		cis-Thujopsene	Sesquiterpenoids	C_15_H_24_	470-40–6	0.42	0.20	-	-
16	20.18		2,6,10-Trimethyltridecane	Aliphatic alkanes	C_16_H_34_	3,891-99–4	1.62	1.88	-	-
17	20.75		1-Dodecanol	Fatty alcohols	C_12_H_26_O	112-53–8	-	-	0.35	-
18	21.14		α-Curcumene	Sesquiterpenoids	C_15_H_22_	644-30–4	-	0.23	-	-
19	21.71		Pentadecane	Aliphatic alkanes	C_15_H_32_	629-62–9	1.94	2.05	0.75	0.26
20	22.16		α-Farnesene	Sesquiterpenoids	C_15_H_24_	502-61–4	2.15	5.33	3.97	2.17
21	25.13		Nerolidol	Sesquiterpenoids	C_15_H_26_O	7212-44–4	-	-	0.83	-
22	27.20		Hexadecane	Aliphatic alkanes	C_16_H_34_	544-76–3	1.90	2.10	1.91	1.02
23	27.59		Cedrol	Sesquiterpenoids	C_15_H_26_O	77-53–2	0.92	1.05	0.93	0.32
24	33.11		Heptadecane	Aliphatic alkanes	C_19_H_40_	629-92–5	0.45	0.74	1.24	1.09
25	35.45		α-Hexylcinnamaldehyde	Aromatic aldehydes	C_15_H_20_O	101-86–0	-	-	-	0.90
26	36.24		Benzyl benzoate	Aromatic esters	C_14_H_12_O_2_	120-51–4	-	0.75	2.27	3.54
27	39.53		Neophytadiene	Diterpenoids	C_20_H_38_	504-96–1	-	0.56	2.37	3.50
28	39.78		Hexahydrofarnesyl acetone	Sesquiterpenoids	C_18_H_36_O	502-69–2	3.15	5.44	9.17	8.02
29	40.57		9-Eicosyne	Aliphatic alkynes	C_20_H_38_	71899-38–2	-	0.14	0.85	1.16
30	42.11		2,6,10-Trimethylpentadecane	Sesquiterpenoids	C_18_H_38_	3,892-00–0	-	-	0.73	0.97
31	44.49		Isophytol	Diterpenoids	C_20_H_40_O	505-32–8	0.60	1.12	8.83	14.09
32	52.41		Heneicosane	Aliphatic alkanes	C_21_H_44_	629-94–7	-	0.36	2.54	3.60
33	52.70		Phytol	Diterpenoids	C_20_H_40_O	150-86–7	-	-	0.83	2.16
34	55.17		Tetracosane	Aliphatic alkanes	C_24_H_50_	646-31–1	-	-	0.45	0.64
Total amounts of the identified compounds							52.13	38.79	45.70	49.98
Total numbers of compounds							19	25	26	25

As shown in Table 3, when the extraction temperature rose from 50°C to 90°C in the HS-SPME procedure, the temperature changes obviously influenced the component profiles. With the increase in extraction temperature, more and more compounds were detected. The quantities of the compounds increased from 19 to 26, whereas at 100°C, the quantities of the compounds did not show obvious changes compared with those at 90°C, which indicated that most of the volatile and semi-volatile compounds in *J. grandiflorum* L. could be extracted under 90°C. Obviously, the compounds with small molecular weight could reach the maximum extraction amounts at 50°C and 70°C, such as phenylethyl alcohol (C_7_H_8_O) up to 8.01% at 50°C and down to 1.70% at 100°C. Some compounds such as levomenthol (C_10_H_20_O) amounted to 6.29% and 1.43% at 50°C and 70°C, respectively, while they could not be detected at 90°C and 100°C. Even decanal (C_10_H_20_O) only was detected at 50°C. Contrarily, the contents of compounds with large molecular weight increased with the increase in extraction temperature, such as two diterpenoid compounds, neophytadiene (C_20_H_38_, amounting to 0% at 50°C and 3.50% at 100°C, respectively) and isophytol (C_20_H_40_O, amounting to 0.60% at 50°C and 14.09% at 100°C, respectively). Additionally, in this study, the relative amounts of most volatile compounds except phytol were greater than those in essential oil extracted using steam distillation (Wei et al., 2015). The comparative analysis of the two results further indicates that steam distillation could decrease volatile compounds with a small molecular weight, which is identical to the findings at different extraction temperatures in this study, and also indicates that HS-SPME could obtain more true volatile compound profiles of aromatic herbs.

### 3.2 Network pharmacology prediction

#### 3.2.1 Target prediction of volatile compounds inhibiting inflammation and neuroinflammation

**FIGURE 2:**
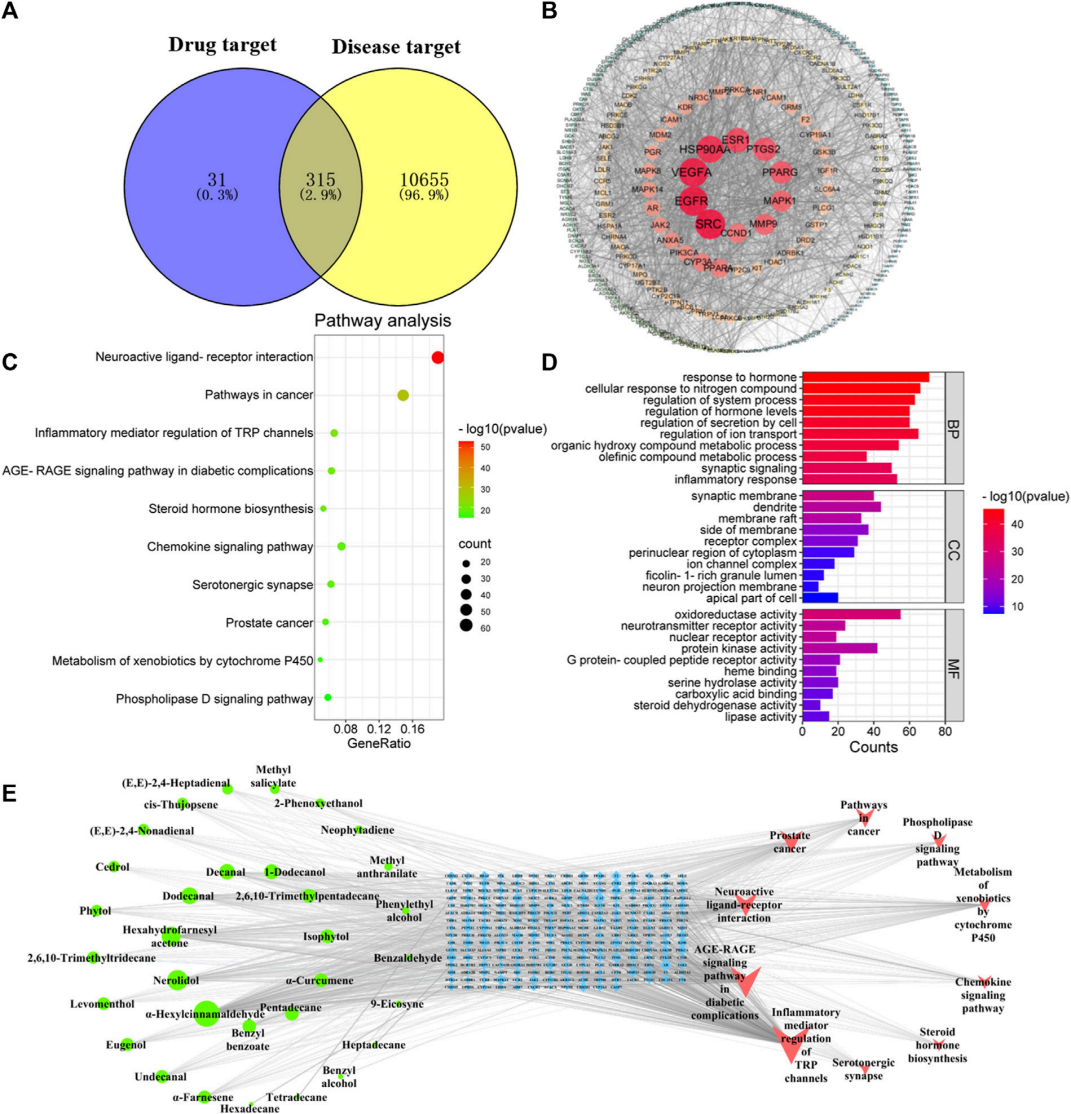
Network pharmacology predicted the possible compound–target–pathway interaction in JGEO-mediating inflammation. **(A)** Venn analysis on putative targets of JGEO as well as inflammation-related genes. **(B)** PPI of 346 targets of JGEO associated with inflammation. **(C)** Bubble chart of KEGG pathway analysis. Top 10 pathways with corresponding *p*-values are displayed in a dot plot. The sizes of the dots represent the gene count of each term, and the color scales indicate the *p*-values. **(D)** GO enrichment analysis. The length and color of the bands characterize the number of targets involved in the related biological processes. **(E)** Construction of the key compound–target–pathway. The network depicts the relationships among compounds, targets, and pathways. The green circle represents the active compounds, the blue square in the middle is for the core targets, and the red triangle is for the pathway.

A total of 346 targets related to volatile compounds in *Jasminum grandiflorum* L. were searched by the Swiss TargetPrediction database. For neuroinflammation-related targets and inflammation-related targets, 788 *Homo sapiens* targets and 10,950 *H. sapiens* targets were screened by GeneCards, respectively. A total of 10,970 inflammatory target genes were finally obtained by eliminating duplicate target genes. By inputting the abovementioned targets into Venny 2.1.0 software, as shown in Figure 2A, 315 intersection targets between compounds with inflammation and neuroinflammation were obtained, respectively.

#### 3.2.2 Protein–protein interaction network

Based on the intersection targets, PPI networks of targets related to volatile compounds and targets related to inflammation were constructed by importing them into Cytospace 3.6.0 software. There were 315 nodes with 2,903 connections in the PPI networks, as shown in Figure 2B. Each circular node in the figure represents each intersection target, the node size and color depth show the degree value, and the edge thickness shows the combined score. Meanwhile, the more central the node is, the higher the degree value is. The PPI network was divided by the node degree values of 1–19, 20–32, 33–57, and 58–89 (shown in Figure 2B), respectively.

#### 3.2.3 GO and KEGG pathway enrichment analyses

In order to explore the potential mechanism of volatile compounds mediating inflammation, a KEGG pathway enrichment bubble chart was constructed. As shown in Figures 2A, C, the number of inflammation-related signaling pathways, such as neuroactive ligand–receptor interaction, inflammatory mediator regulation of TRP channels, AGE-RAGE signaling pathway in diabetic complications, and steroid hormone biosynthesis, especially the neuroactive ligand–receptor interaction signal pathway, is the most significant.

GO enrichment analysis of 315 intersection targets between compounds with inflammation was performed to clarify the possible role of candidate targets. The results of GO enrichment analysis (Figure 2D) indicated that the key targets were mainly involved in the response to hormones, cellular response to the nitrogen compound, regulation of the system process, regulation of hormone levels, and other biological processes. Synaptic membrane, dendrite, membrane raft, and other cellular components were highly related. Molecular function included oxidoreductase activity, neurotransmitter receptor activity, nuclear receptor activity, and others.

#### 3.2.4 Compound–target–pathway networks

In order to further obtain the key nodes of the interaction between compounds and inflammation, a network diagram with 278 nodes with 1,203 connections, including 10 pathway nodes, 236 target nodes, and 32 compound nodes, was obtained. As shown in Figure 2E, the higher the degree value, the more important the node, the greater the intermediary centrality of the node, the greater the probability that the node is the core node, the greater the compactness and centrality of the node, and the closer the distance between this node and other nodes. This means that the more important the node is, the compounds with the top five degree values were α-hexylcinnamaldehyde, nerolidol, hexahydrofamesyl acetone, dodecanal, and decanal, which means these five compounds are relatively important.

#### 3.2.5 Molecular docking

**FIGURE 3:**
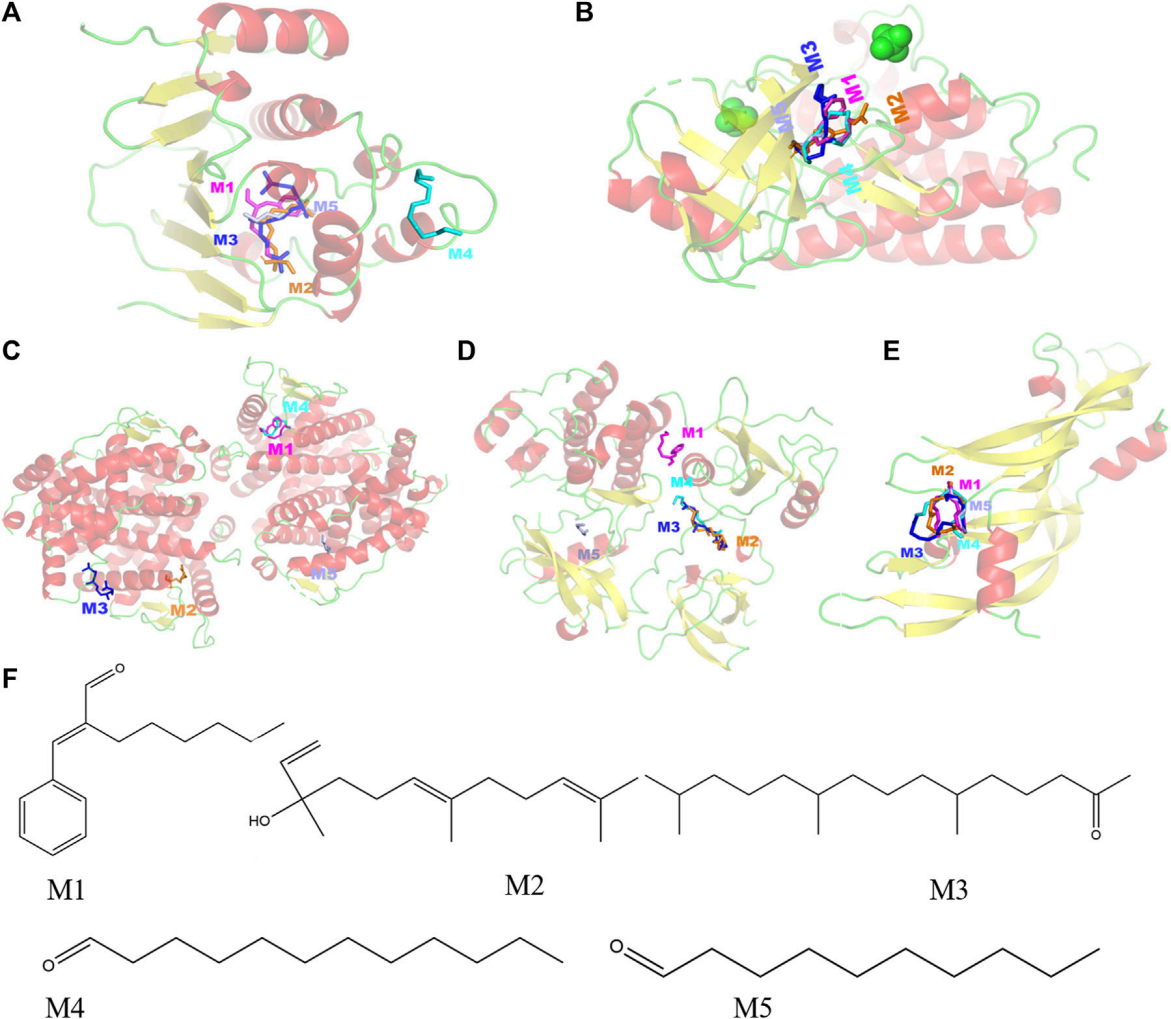
Molecular models of five key compounds binding to their predicted protein targets. **(A)** HSP90AA1, **(B)** EGFR, **(C)** ESR1, **(D)** SRC, **(E)** VEGFA, and **(F)** chemical structures of the five key compounds. M1: α-hexylcinnamaldehyde, M2: nerolidol, M3: hexahydrofarnesyl acetone, M4: dodecanal, and M5: decanal.

Selecting the compounds with the top five degree values in compound–target–pathway networks and targets with the top five degree values in the PPI network, which were conjugated and scored by molecular simulation software (AutoDockTools), based on that the lower the binding energy, the higher the affinity between the receptor and the ligand and the higher the possibility of the interaction between compounds and targets, the targets, including SRC(PDB ID: 1FMK), EGFR (PDB ID:5UG9), VEGFA (PDB ID:1MKK), HSP90AA1(PDB ID:7S9I), and ESR1(PDB ID:3OS8), were predicted as the key targets of the key compounds in that the docking binding energies of the key compounds and the key targets were less than −3.9 kcal/mol, as shown in Table 4 and Figure 3. Especially, these five target proteins are all related to inflammatory mediator regulation of transient receptor potential (TRP) channels. TRP channels, as a superfamily of non-selective cation channels, are involved in the progression of neurodegenerative disorders (such as Alzheimer’s and Parkinson’s diseases) (Silverman et al., 2020). Additionally, the results also indicate that JGEO could exhibit inhibitory effects on inflammation via multi-compound and multi-target action modes.

**TABLE 4:** Binding energies of five key compounds binding to their predicted protein targets.

Key compounds	Key targets	PDB ID	Binding energies/(kJ/mol)
α-Hexylcinnamaldehyde	HSP90AA1	7S9I	−8.1
Nerolidol	HSP90AA1	7S9I	−7.9
Hexahydrofarnesyl acetone	HSP90AA1	7S9I	−7.5
Nerolidol	EGFR	5UG9	−7.1
Nerolidol	ESR1	3OS8	−7.1
Hexahydrofarnesyl acetone	EGFR	5UG9	−6.9
α-Hexylcinnamaldehyde	EGFR	5UG9	−6.6
Nerolidol	SRC	1FMK	−6.2
α-Hexylcinnamaldehyde	SRC	1FMK	−5.8
α-Hexylcinnamaldehyde	VEGFA	1MKK	−5.8
Hexahydrofarnesyl acetone	SRC	1FMK	−5.8
Decanal	HSP90AA1	7S9I	−5.6
Dodecanal	HSP90AA1	7S9I	−5.4
Dodecanal	ESR1	3OS8	−5.4
Nerolidol	VEGFA	1MKK	−5.3
α-Hexylcinnamaldehyde	ESR1	3OS8	−5.2
Hexahydrofarnesyl acetone	ESR1	3OS8	−5.2
Hexahydrofarnesyl acetone	VEGFA	1MKK	−5.1
Dodecanal	EGFR	5UG9	−5.1
Decanal	EGFR	5UG9	−5.0
Decanal	ESR1	3OS8	−4.4
Dodecanal	SRC	1FMK	−4.3
Decanal	SRC	1FMK	−4.1
Dodecanal	VEGFA	1MKK	−3.9
Decanal	VEGFA	1MKK	−3.9

### 3.3 The effects of *Jasminum grandiflorum* L. essential oil on BV-2 cell viability and morphology

**FIGURE 4:**
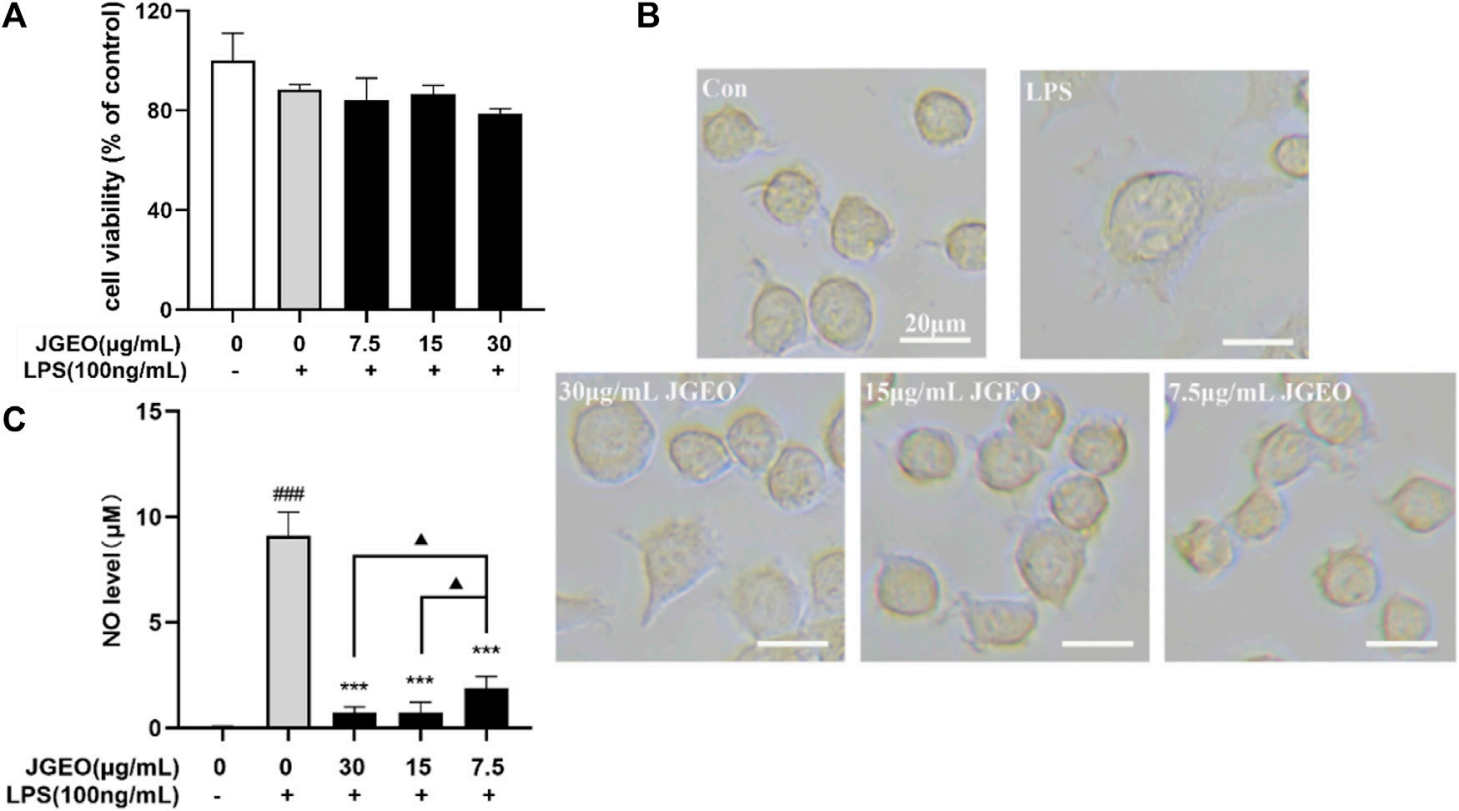
Effects of JGEO on BV-2 cell viability, morphology, and NO level. BV-2 cells were exposed to JGEO at 7.5, 15, and 30 μg/mL for 4 h and treated with 100 ng/mL of LPS for 24 h, respectively. **(A)** The cell viability was evaluated by CCK8 assay. All treated groups had no significant difference compared to the control group (*p >* 0.05). **(B)** The cell morphologies were observed under a light microscope. BV-2 cells in the control group were quiescent with small soma and few pseudopodia, while those in the LPS-treated model group displayed enlarged soma and short dendritic arbors like amoeba, while JGEO reversed the morphological changes induced by LPS. **(C)** NO levels were determined by Griess reaction. All JGEO-pretreated groups significantly decreased the NO level. Data were analyzed by statistical analysis following the procedure mentioned in“Section 2.8” (^###^ *p <* 0.001 vs. the control group, ***p < 0.001 vs. the LPS model group, and ^▲^ *p <* 0.05 in the interaction of the two groups).

To examine the potential cytotoxic effect on BV-2 cells, BV-2 cells were incubated with 7.5, 15, and 30 μg/mL JGEOs for 4 h, respectively, and then stimulated with 100 ng/mL of lipopolysaccharide for 24 h. The cell viability was evaluated by CCK8 assay. As shown in Figure 4A, BV-2 cell viability treated with JGEO and LPS was not significantly influenced compared to the control group (*p >* 0.05), which indicated that 100 ng/mL LPS stimulation and 7.5–30 μg/mL JGEO treatment did not exert cytotoxicity. Therefore, JGEO at these concentrations was used for the subsequent studies.

Considering that LPS could stimulate the morphological changes of the microglia (Chuang et al., 2015), the microglial morphologies were observed under a light microscope. As shown in Figure 4B, BV-2 cells in the control group were quiescent with small soma and few pseudopodia, while those in the LPS-treated group emerged the enlarged soma and short dendritic arbors like amoeba, which indicated 100 ng/mL of LPS-activated BV-2 cells (Nimmerjahn et al., 2005), while the JGEO-treated BV-2 cells showed a morphology resembling closely to unstimulated ramified microglia in the control group, which indicated that JGEO suppressed the activation of LPS-induced BV-2 cells after pretreating for 4 h.

### 3.4 *Jasminum grandiflorum* L. essential oil suppressed NO production

The activated microglia would express the NADPH oxidase, which generates inducible nitric oxidase and then converts arginase into NO. NO is an unstable messenger molecule, which immediately converts to stable molecules of nitrite and nitrate. Increasing NO to a certain amount within the cells leads to induction of apoptosis; however, overproduction of NO may cause many disorders, such as neurologic disorders (Murphy, 1999). To evaluate the abilities of JGEO suppressing microglial activation, the effects of JGEO on suppressing LPS-induced NO excessive release were carried out. As shown in Figure 4C, 100 ng/mL LPS stimulation markedly induced NO release compared to the control group (*p <* 0.001), which also indicated that the LPS-induced BV-2 activation model was successful. In addition, 7.5–30 μg/of JGEO significantly decreased NO levels of LPS-induced BV-2 cells in a dose-dependent manner compared to the LPS group (*p <* 0.001). The results indicated that JGEO could exhibit anti-neuroinflammatory effects by suppressing NO production of overactivation microglia.

### 3.5 *Jasminum grandiflorum* L. essential oil inhibited the levels of TNF-α and IL-1β

**FIGURE 5:**
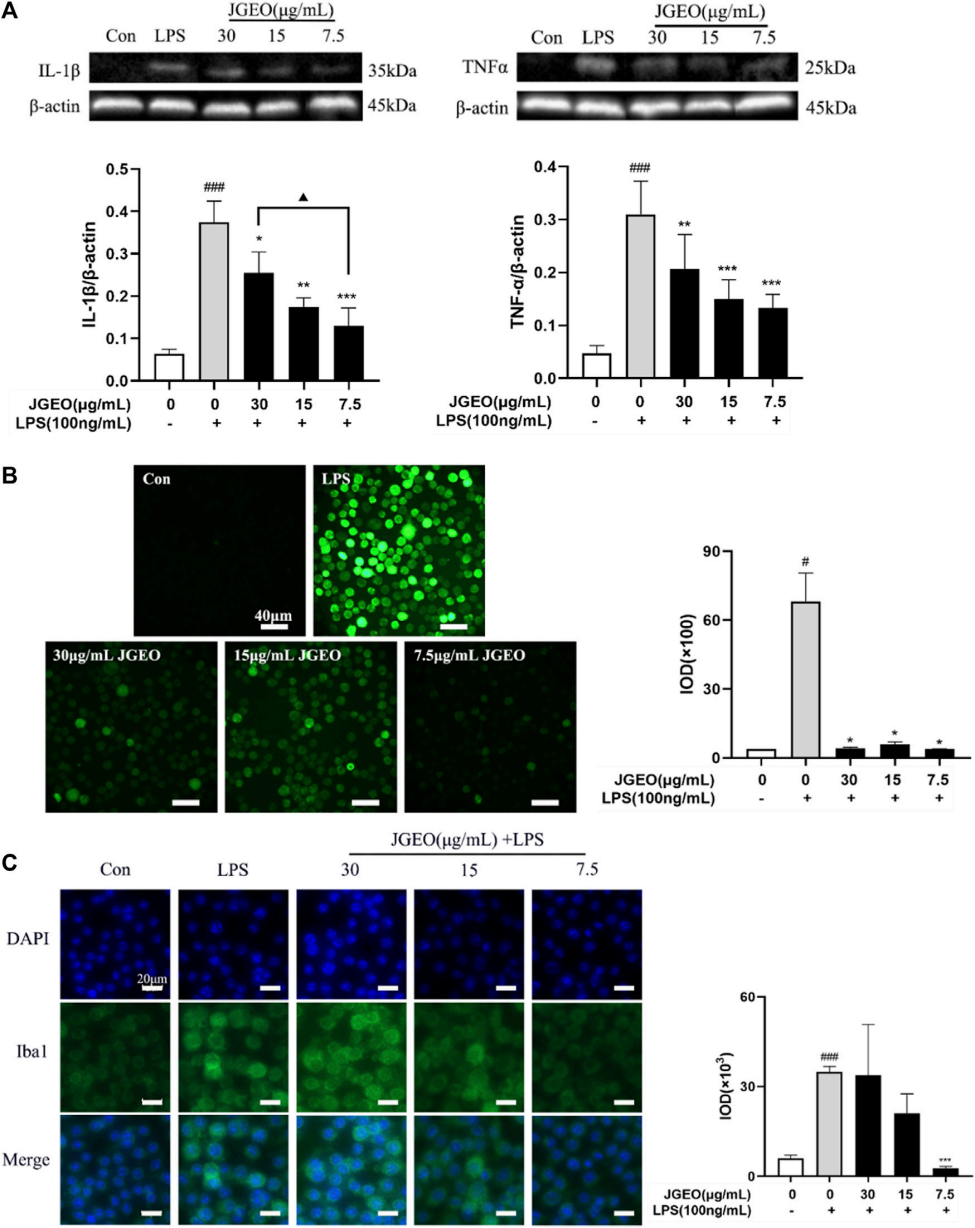
Effects of JGEO on LPS-induced BV-2 cells. Con, the control group. BV-2 cultures were exposed to DMEM. LPS, the model group. BV-2 cultures were exposed to DMEM for 4 h and then treated with 100 ng/mL of LPS for 24 h. BV-2 cells were exposed to JGEO at 30, 15, and 7.5 μg/mL for 4 h and then treated with 100 ng/mL of LPS for 24 h, respectively. **(A)** JGEO decreased IL-1β and TNF-α levels of LPS-induced BV-2 cells. **(B)** JGEO decreased the ROS level of LPS-induced BV-2 cells. Cells in all groups were incubated with DCFH-DA for 1 h at 37°C in the dark and imaged using a LEICA DMi8 microscope, and the IOD (integral optical density) of ROS fluorescence intensity per field was calculated by the ImageJ software. **(C)** JGEO regulated LPS-induced BV-2 cell activation. Cells were imaged using a LEICA DMi8 microscope, and the IOD of anti-Iba1 green fluorescence intensity per field was calculated by the ImageJ software. Data were analyzed by statistical analysis following the procedure mentioned in “Section 2.8” (^#^ *p* < 0.05, ^###^ *p <* 0.001 vs. the control group, ^*^ *p <* 0.05, ^**^ *p <* 0.01, and ^***^ *p <* 0.001 vs. the model group, and ^▲^ *p <* 0.05 in the interaction of the two groups).

The activated microglia would produce proinflammatory cytokines, especially TNF-α and IL-1β (Colonna and Butovsky., 2017). Though microglial activation is necessary and critical for host defense, overactivation of microglia is neurotoxic (Leng and Edison., 2021). Here, we used TNF-α and IL-1β as the index by Western blotting assay to evaluate the anti-inflammatory effects of JGEOs. As shown in Figure 5A, LPS-induced BV-2 cells significantly increased the levels of TNF-α and IL-1β compared to the control group (*p <* 0.001), while the production of TNF-α and IL-1β was strongly inhibited by JGEO pretreatment at 7.5–30 μg/mL concentrations compared to the LPS model group (*p <* 0.05). The results indicated that JGEO exhibited significant anti-neuroinflammatory effects by suppressing the production of TNF-α and IL-1β of overactivation microglia.

### 3.6 *Jasminum grandiflorum* L. essential oil inhibited ROS accumulation

Oxidative stress contributes significantly to the early onset and progression of neurodegenerative diseases, thus eliminating the oxidative stress cascade to correct the abnormal microenvironment of the CNS has been proven as a new perspective for treating neurodegenerative diseases (Chuang et al., 2015). ROS, as a typical feature of oxidative stress and a key initiator of neuronal damage, is commonly used as an index to evaluate the neuroprotective effects of experimental samples. The inflammatory response of microglial cells to LPS was associated with a robust elevation of intracellular oxidative stress (Dos-Santos-Pereira et al., 2020). As shown in Figure 5B, intensities of green fluorescence of DCF from DCFH-DA among the groups were significantly different. The stronger the green fluorescence, the greater the ROS accumulation. To further compare the changes of ROS accumulation, the intensities of the green fluorescence were analyzed by ImageJ. The results showed that 100 ng/mL of LPS significantly increased the ROS level of BV-2 cells compared to the control group (*p <* 0.05), while 7.5–30 μg/mL of JGEO significantly decreased the ROS level compared to the LPS model group (*p <* 0.05), which indicated that JGEO significantly reduced the release of ROS in LPS-induced BV-2 cells. The results indicated that *Jasminum grandiflorum* L. essential oil could exhibit neuroprotective effects by inhibiting the intracellular accumulation of ROS in overactivation microglia induced by LPS.

## 4 Conclusion

In this study, a total of 34 volatile compounds with clearly different volatilities in *Jasminum grandiflorum* L. flowers were detected, which will be helpful to choose a reasonable drying method. Network pharmacological analysis forecasted that α-hexylcinnamaldehyde, nerolidol, hexahydrofarnesyl acetone, dodecanal, and decanal were the top five key compounds, and SRC, EGFR, VEGFA, HSP90AA1, and ESR1 were the top five key targets; meanwhile, the binding energies between them were less than −3.9 kcal/mol. Additionally, these five target proteins are all related to inflammatory mediator regulation of TRP channels, which is involved in the progression of neurodegenerative disorders (such as Alzheimer’s and Parkinson’s diseases) (Silverman et al., 2020). The results also indicate that JGEO could exhibit inhibitory effects on inflammation via multi-compound and multi-target action modes. In the LPS-induced microglial cell model, JGEO not only clearly reversed the topographical changes of BV-2 cells but also significantly inhibited the production of NO and ROS and suppressed the expressions of TNF-α, IL-1β, and Iba1, which verified the network pharmacological analysis at some extent. Taken together, the results indicate that JGEO reduces the neuroinflammatory and oxidative stress responses by suppressing microglia activation, which could provide some basis for the traditional use of JGEO in treating neuroinflammation-related disorders. While considering the species-specific differences between human and rodent microglia, the inhibitory effects of JGEO on microglia activation need to be evaluated further using microglia cells from human or AD animals.

## Data Availability

The original contributions presented in the study are included in the article/Supplementary Material; further inquiries can be directed to the corresponding author.

## Abbreviations

AD, Alzheimer’s disease; CNS, central nervous system; CAR/PDMS, carboxen/polydimethylsiloxane; GC–MS, gas chromatography–mass spectrometry; HS-SPME, headspace solid-phase microextraction; Iba1, ionized calcium-binding adapter molecule 1; IL-1β, interleukin-1β; JGEO, *Jasminum grandiflorum* L. essential oil; LPS, *Escherichia coli* lipopolysaccharide; NO, nitric oxide; ROS, reactive oxygen species; and TNF-α, tumor necrosis factor-α.
